# Pedicled Myocutaneous Flap Breast Reconstruction in the Community Hospital Setting: An Analysis of Cost and Complications

**DOI:** 10.7759/cureus.11806

**Published:** 2020-11-30

**Authors:** Zachary Long, Franziska Huettner, Amy Kells

**Affiliations:** 1 Plastic and Reconstructive Surgery, St. Barnabas Hospital, Bronx, USA; 2 Plastic and Reconstructive Surgery, Berkshire Health System, Pittsfield, USA; 3 Plastic and Reconstructive Surgery, Washington University, St. Louis, USA

**Keywords:** plastic surgery, reconstructive surgery, breast surgery, breast reconstruction, breast cancer, pedicled myocutaneous flap, community hospital

## Abstract

Background

Free tissue transfer breast reconstruction is an option for breast cancer patients that is precluded by a number of factors. The authors’ objective was to assess the use of pedicled myocutaneous breast reconstruction in the community hospital setting, with more limited resources, as a viable option with comparable rates of complications, cost, and outcomes.

Methods

The authors performed a retrospective cohort review of pedicled myocutaneous breast reconstructions of a single surgeon at a community-based institution from 2015 to 2019. Rates of complications, including partial and total flap failure, infection, seroma/hematoma, and reoperation were evaluated, as well as initial hospital cost, readmission cost, and subjective patient satisfaction. Statistical analysis was performed on the data and compared to published data on free flap breast reconstruction with regards to similar data points.

Results

There were ten patients included in the analysis. This data demonstrated an immediate reoperation rate of 0%, with no incidence of partial or total flap loss, infection, seroma, hematoma, or medical complication. Delayed complications included delayed wound healing of the donor site (10%), abdominal wall bulge (10%), and umbilical partial necrosis (10%). The average length of initial stay was 5.7 days and the average initial hospital costs were $94,717.

Conclusions

As demonstrated at St. Barnabas Hospital, this type of breast reconstruction does not require the presence of a microsurgery fellowship program, high volumes, significant ancillary staff training, or other costly resources to monitor the patient, yet yields comparable or favorable rates of complications when compared to free tissue reconstruction. This allows more reconstructive options to be available to patients who may not have access to large tertiary centers for free flap reconstruction.

## Introduction

Breast cancer is the most frequently diagnosed life-threatening cancer and the leading cause of cancer deaths in women in the world [1]. Most breast cancers require significant surgical intervention that often leads to an acquired absence of breast tissue. Plastic surgeons are frequently tasked with providing soft tissue reconstruction of the breasts. There are many options available, but the likelihood that a breast cancer patient will undergo reconstruction by a plastic surgeon is directly correlated with the availability of resources (plastic surgeon, tertiary center, etc.) within his or her community [2].

Pedicled myocutaneous flaps have been used in breast reconstruction since the 1980’s when Hartrampf, Scheflan, and Black introduced it in 1982 [3]. They are often cited as a more reliable option than free tissue transfer for breast reconstruction due to their reliable vascular source that is left in continuity. It can also be performed by many plastic surgeons that have had exposure to pedicled flap breast reconstruction in training. In contrast, due to the nature of free tissue transfer, they are associated with higher cost, more required hospital resources, and higher rates of complications [4].

To date, there is little published studies that evaluate the specific differences between free tissue transfer and pedicled flap breast reconstruction in the community hospital setting with regards to complications, hospital cost, and patient satisfaction. Golpanian et al. reviewed national inpatient data comparing free and pedicled transverse rectus abdominis myocutaneous (TRAM) flap reconstruction and found a significantly increased rate of complications, total cost, and length of stay for those undergoing free flap reconstruction [5-7]. However, the goal of this research was to elucidate if the same applied specifically in the community-based setting. This retrospective review is aimed at quantifying factors that differentiate pedicled flap breast reconstruction at St. Barnabas Hospital and published national data on free flap breast reconstruction in order to highlight a more accessible option for patients. In doing so, this will give evidence that a successful breast reconstruction can be offered to more patients, even within smaller community hospital settings with more limited resources and staff training.

## Materials and methods

We designed a retrospective cohort review study and obtained IRB approval on April 23, 2019 (study number 2019.18). The operating room records from the senior author between 2015 to 2019 at St. Barnabas Hospital in the Bronx, NY were evaluated. These records were then filtered by type of procedure performed to include pedicled TRAM breast reconstruction. The inclusion criteria were any patients that underwent immediate breast reconstruction after mastectomy with a pedicled myocutaneous flap. Exclusion criteria included any patients with incomplete records.

Postoperative medical complications were defined as diagnosis of pulmonary embolus (PE), deep venous thrombosis (DVT), or myocardial infarction (MI) within 30 days of procedure. Other postoperative complications, such as immediate reoperation rate, delayed wound healing of flap, partial or full-thickness flap necrosis, seroma, hematoma, use of drains, and donor site morbidities were also documented. Finally, initial hospital length of stay, need for readmission (with associated length of stay), and hospital costs were evaluated. Subjective patient satisfaction was determined by documentation in both inpatient and outpatient notes. Individual patient comorbidities were noted, including age, body mass index (BMI), hypertension, diabetes (with associated Hemoglobin A1c, if applicable), anticoagulant use, steroid use, diagnosis of breast cancer, hormonal therapy, pre- or post-operative chemotherapy, pre- or post-operative radiation therapy, bleeding disorders, and psychiatric disorders. However, analysis of these demographics were not the goal of our study and were only documented for informational purposes.

Cross tabulation was then performed across the incidences of complications, hospital length of stay, and hospital cost. These data point values were then compared to corresponding rates of nationally published data on free tissue transfer breast reconstruction.

## Results

A total of 10 patients were included in the retrospective review. With regards to immediate reoperation, which was defined as procedures performed in the operating room during the same admission, there was one patient who had to return for axillary lymph node dissection. However, there were no cases of immediate reoperation for flap- or donor site-related complications. 

Four patients required readmission (within three months of initial operation). There were two cases of irrigation and debridement of the donor site with vacuum-assisted wound closure device (VAC) applications. There was also one case each of ventral hernia repair and scar revision (for abdominal wall bulge) and contralateral mastectomy for breast cancer. Removing the patient who had a contralateral mastectomy, as this was due to the nature of the patient’s pathology, and not a sequelae of the initial procedure, our institution’s data demonstrated an improved delayed case reoperation rate of 30% (Table 1). The patient who experienced delayed wound healing of the donor site was treated with local wound care without further complication.

**Table 1:** Summary of complications *Patient underwent axillary lymph node dissection; +Includes one patient that underwent contralateral mastectomy for new onset breast cancer. PE, pulmonary embolus; DVT, deep venous thrombosis; MI, myocardial infarction.

Complication	Number of patients
Immediate reoperation	1*
Delayed reoperation	4^+^
Flap delayed wound healing	0
Partial or total flap necrosis	0
Seroma	0
Hematoma	0
Infection	0
Abdominal necrosis	2
Abdominal bulge	1
Umbilical necrosis	0
Abdominal delayed wound healing	1
PE/DVT/MI	0

There were no patients who experienced partial or total flap necrosis, hematoma, or seroma. All patients underwent closed system drainage of their donor sites and breast pockets. Analysis of medical postoperative complications yielded no patients experiencing venous thrombotic event, myocardial infarction, or cerebrovascular event.

With regards to hospital length of stay and costs, our patients had an average initial length of stay of 5.7 days, with an overall range of four to nine days. The patient who had a nine-day hospital stay required an additional axillary lymph node dissection that delayed her discharge. The other patients who had hospital stays greater than five days were delayed in discharge due to pain control and glycemic control. When analyzing initial hospital costs, the average cost was $94,717, with an overall range of $73,098 to $114,746. Of the four patients that required readmission, their readmission length of stays were on average four days, ranging between one and seven days. Their readmission hospital costs ranged between $12,149 and $22,114, with an overall average of $13,240 (Table 2).

**Table 2:** Summary of patient demographics BMI, body mass index; LOS, length of stay; USD, United States dollars.

Patient Demographics	Description
Total patients	10
Average age, years	50.9
Average BMI	29.2
Breast cancer diagnosis	9
History of tobacco use	2
Hypertension	6
Diabetes	2
Steroid use	0
Hormonal therapy	5
Preoperative chemotherapy	3
Postoperative chemotherapy	3
Preoperative radiation	3
Postoperative radiation	2
Psychiatric disorder	3
Average initial hospital LOS, days	5.7
Average initial hospital cost, USD	$94,717
Average readmission LOS, days	4
Average readmission hospital cost, USD	$13,240

## Discussion

The data here represents a comprehensive single-surgeon experience of pedicled TRAM breast reconstruction at an inner-city community hospital. The analysis is significant because of its representation of a smaller, low-volume hospital with very limited resources, especially when compared to large tertiary, academic centers with higher volumes. We demonstrated a 0% immediate reoperation rate, a delayed reoperation rate of 30%, average initial hospital length of stay of 5.7 days, and an average initial hospital cost of $94,717.

There is little literature that directly compares a community hospital’s experience with pedicled myocutaneous breast reconstruction to previously published data on free tissue transfer at larger institutions. One of the goals of this study was to demonstrate that with more limited resources, including hospital equipment, ancillary staff training, and lower volumes, pedicled flap reconstruction can provide a larger patient population options that are comparable or better in complication profile, and postoperative satisfaction. This allows those patients to stay closer to home during what can be very challenging times.

Comprehensive autologous free tissue breast reconstruction literature from the University of Pennsylvania demonstrated rates of immediate major surgical complications, delayed major surgical complications, minor surgical complications, and medical complications as 4%, 6.4%, 47.6%, and 5.9%, respectively [8]. Comparatively, this data demonstrated no immediate major surgical or medical complications. Their rate of hernia/bulge was 3.4%, which was lower than this data, which demonstrated one patient (7%). However, their data also demonstrated a rate of complete flap loss of 4.2%, while there were no instances at this institution. Additionally, they showed rates of infection, seroma, delayed breast healing, and delayed donor site healing as 7.5%, 3.9%, 32%, and 18%, respectively. Within this patient set, there were no instances of infection, seroma, or flap complication, and the rate of delayed donor site healing was 10%. Even compared to a large retrospective study of 252 ipsilateral pedicled TRAM reconstructions, Clugston et al. demonstrated rates of mastectomy flap necrosis, partial flap necrosis, abdominal flap necrosis, seroma, bulge, hematoma, and DVT/PE of 9.4%, 2%, 10.5%, 9.5%, 5.8%, 1.6%, and 1.1%, respectively [9]. This data showed improved rates of these complications. 

Hospital length of stay and total cost were more significant at this institution, when compared to other nationally published data. According to data from Billig et al. the median national cost for autologous free flap breast reconstruction was $22,677 and the median length of stay was four days [10]. Fischer et al. demonstrated an average total cost of $19,106 in those without, to $28,261 in those with surgical complications [8]. In contrast, the average initial length of stay for this data was 5.7 days and the average hospital cost $94,717. However, one variable that was not delineated in the financial data from this institution was hospital costs for the reconstructive aspect alone. All hospital costs included the reconstructive portion, oncologic procedures and care, and social services provided. Since many cases within the published data on free tissue transfer were delayed, thereby negating the oncologic resection costs, it is difficult to directly compare the hospital costs, as all of this institution’s cases were with immediate reconstruction.

Additionally, chart review demonstrated subjective patient satisfaction with the pedicled breast reconstruction from every patient. There were documented improvements in patient’s perception of both breast shape and abdominal contour. However, this is based on medical documentation, not standardized surveys, making it difficult to quantify a significance other than anecdotal. Another limitation of this study was the lower power, when compared to nationally published data. This can help direct future research into including multiple community-based hospitals, which would improve consistency in comparisons. 

## Conclusions

As demonstrated at St. Barnabas Hospital, this type of breast reconstruction does not require the presence of a microsurgery fellowship program, high volumes, significant ancillary staff training, or other costly resources to monitor the patient, yet yields comparable or favorable rates of complications when compared to free tissue reconstruction. As this hospital is representative of many community hospitals with lower volumes, this allows more reconstructive options to be available to patients who may not have access to large tertiary centers for free flap reconstruction, which has been shown to have higher rates of complications and cost in low-volume centers. While this data demonstrates higher hospital costs than nationally published free tissue transfer data, future analysis of the specific costs of breast reconstruction could be analyzed to reveal a better comparison.
